# Preoperative clinical-radiomics nomogram for microvascular invasion prediction in hepatocellular carcinoma using $^{18}$F-FDG PET/CT

**DOI:** 10.1186/s12880-022-00796-4

**Published:** 2022-04-15

**Authors:** Yutao Wang, Shuying Luo, Gehui Jin, Randi Fu, Zhongfei Yu, Jian Zhang

**Affiliations:** 1 The Affiliated Hospital of Medical School, Ningbo University, Ningbo, Zhejiang Province 315020 China; 2 Faculty of Electrical Engineering and Computer Science, Ningbo University, Ningbo, Zhejiang Province 315211 China; 3 Medical School, Ningbo University, Ningbo, Zhejiang Province 315211 China; 4 Shanghai Universal Medical Imaging Diagnostic Center, Shanghai University, Building 8, 406 Guilin Road, Xuhui District, Shanghai, 201103 China

**Keywords:** Hepatocellular carcinoma, Microvascular invasion, 18F-FDG PET/CT, Radiomics

## Abstract

**Purpose:**

To develop a clinical-radiomics nomogram by incorporating radiomics score and clinical predictors for preoperative prediction of microvascular invasion in hepatocellular carcinoma.

**Methods:**

A total of 97 HCC patients were retrospectively enrolled from Shanghai Universal Medical Imaging Diagnostic Center and Changhai Hospital Affiliated to the Second Military Medical University. 909 CT and 909 PET slicers from 97 HCC patients were divided into a training cohort (N = 637) and a validation cohort (N = 272). Radiomics features were extracted from each CT or PET slicer, and features selection was performed with least absolute shrinkage and selection operator regression and radiomics score was also generated. The clinical-radiomics nomogram was established by integrating radiomics score and clinical predictors, and the performance of the models were evaluated from its discrimination ability, calibration ability, and clinical usefulness.

**Results:**

The radiomics score consisted of 45 selected features, and age, the ratio of maximum to minimum tumor diameter, and $^{18}$F-FDG uptake status were independent predictors of microvascular invasion. The clinical-radiomics nomogram showed better performance for MVI detection (0.890 [0.854, 0.927]) than the clinical nomogram (0.849 [0.804, 0.893]) ($p<0.05$). Both nomograms showed good calibration and the clinical-radiomics nomogram’s clinical practicability outperformed the clinical nomogram.

**Conclusions:**

With the combination of radiomics score and clinical predictors, the clinical-radiomics nomogram can significantly improve the predictive efficacy of microvascular invasion in hepatocellular carcinoma ($p<0.05$) compared with clinical nomogram.

## Introduction

Hepatocellular carcinoma (HCC) is one of the most common malignancy and the fourth leading cause of cancer-related death worldwide [1]. Liver resection and liver transplantation are two universally acknowledged treatments with relatively good prognosis, but the high probability of postoperative tumor recurrence still threatens patients’ long-term survival [2–4]. Therefore, accurate detection of high-risk factors for HCC recurrence preoperatively would play an important role in choosing surgical techniques so as to reduce the chance of HCC recurrence and potentially improve overall survival outcomes.

Microvascular invasion (MVI) refers to the invasion of HCC cells to the peritoneal peritumor tissues. It is one of the pathological features that reflect the aggressiveness of the tumor which can only be seen on the pathological sections under the postoperative microscope. The presence of MVI in HCC patients generally indicates poor survival prognosis, and HCC with MVI also has a much shorter disease-free survival [5]. Therefore, MVI is seen as an important prognostic factor for HCC [6–8]. Due to the extreme heterogeneity of HCC, no stable serological or genomic predictor of MVI has been found so far [9–11]. Recently, preoperative prediction of MVI in HCC using noninvasive imaging modalities has attracted clinical interest. Valuable microscopic features by definition can only be imaged by postoperative biopsy and hence effectively characterizations of the tumor heterogeneity are hard to achieve. Therefore, the preoperative prediction of MVI in HCC remains an urgent problem.

18FDG PET/CT examination is an important method in molecular imaging, in which PET examination provides molecular information such as the function and metabolism of the lesions and CT examination provides precise anatomical localization of the lesions. It can be used for staging and predicting prognosis in patients with malignancy [12]. The maximum standardized uptake value and tumor-to-liver ratio on PET images may be positively correlated with MVI and the prognosis of HCC patients [12, 13]. For HCC patients, primary-tumor FDG avidity is a prognostic indicator of ag-gressive tumor biological behavior and correlates with tumor recurrence [14, 15].

As a new technology, radiomics can improve the assessment and quantification of spatial heterogeneity of tumors by converting medical imaging information into thousands of quantitative features through computer algorithms [16]. At present, some scholars have successfully predicted MVI in HCC preoperatively using radiomics [17–20]. In this study, we used $^{18}$F-FDG PET/CT images for the assessment of MVI of HCC and applied radiomics techniques to establish a radiomics score (Rad-score) from PET/CT images, and we subsequently established a nomogram for the prediction of preoperative MVI. If potential predictors can be identified, 18F-FDG PET/ CT may become an alternative technique that can provide additional information for MVI detection.

## Materials and methods

### Patients

**Fig. 1:**
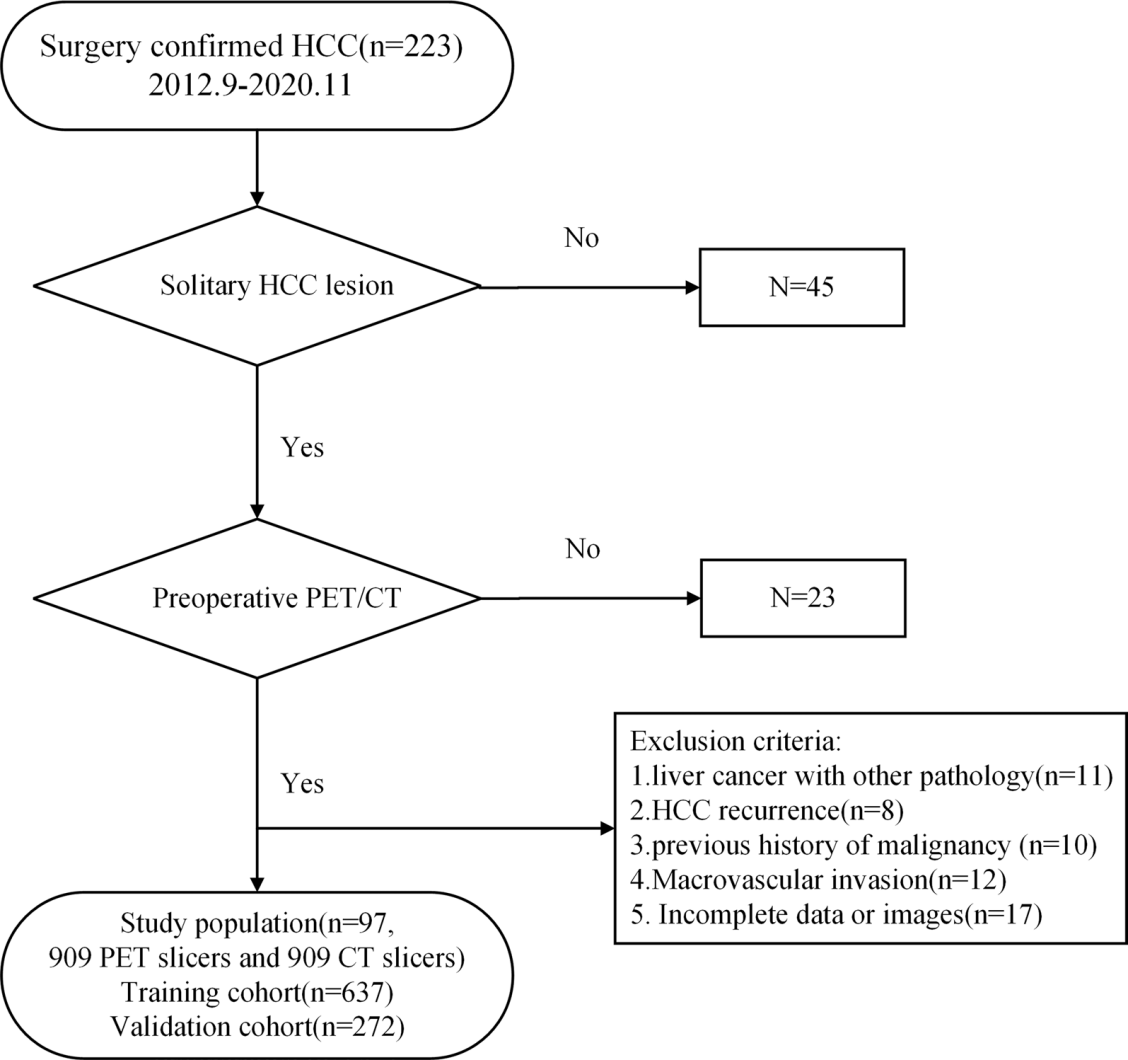
Flow chart of enrolling the study population

In this study, 223 HCC patients’ PET/CT images (from September 2012 to November 2020) from Shanghai Universal Medical Imaging Diagnostic Center and Changhai Hospital Affiliated to the Second Military Medical University were retrospectively analyzed. Among them, the inclusion criteria were as follows: (1) HCC was confirmed by postoperative pathological examination, (2) 18F-FDG PET/CT was performed preoperatively, (3) no invasive examination was performed before 18F-FDG PET/CT examination, (4) imaging studies did not suggest venous tumor thrombus and distant metastasis, and (5) the clinicopathological data were complete. Exclusion criteria were as follows: (1) liver cancer with other pathology, (2) patients with HCC recurrence, (3) previous history of malignancy in the liver or other sites, (4) pathological diagnosis with macroscopic intravascular, and (5) missing clinicopathological data. Figure 1 displays the flow chart of the study population.

After screening, a total of 97 eligible patients were enrolled, 38 had MVI and 59 had no MVI (confirmed by postoperative pathological examination). Depending on the size of the tumor, each patient has a different number of slices. In this paper we obtained 909 PET slicers and 909 CT slicers from 97 cases (including 485 with MVI and 424 without MVI). Each case’s baseline clinical data including age, sex, 18F-FDG uptake status, ratio of maximum to minimum tumor diameter (Max/Min-TD), and tumor location were collected by clinicians with more than 5 years of clinical experience. Clinical information was shown in Table 1. The slicers were randomly divided into a training cohort (N = 637) and a validation cohort (N = 272) at a 7:3 ratio. In a subsequent paper we will deal with a slice as a case for experimentation.

**Table 1:** Clinical characteristics in the training and validation cohorts

Variables	Training cohort (n = 637)		Validation cohort (n = 272)		*p*
	MVI+ (N = 340)	MVI− (N = 297)	MVI+ (N = 145)	MVI− (N = 127)	1.000
Sex, no. (%)					1.000
Male	313 (92.1)	257 (86.5)	130 (89.7)	114 (89.8)	
Female	27 (7.9)	40 (13.5)	15 (10.3)	13 (10.2)	
Age (years), mean ± SD	55.21 ± 11.3	52.68 ± 10.82	54.50 ± 10.80	51.61 ± 10.17	0.270
Max/Min-TD, mean ± SD	3.26 ± 0.82	1.90 ± 0.54	3.07 ± 0.81	2.10 ± 0.67	0.901
18F-FDG uptake status, no. (%)					0.607
Negative	3 (0.9)	108 (36.4)	8 (5.5)	44 (33.6)	
Positive	337 (99.1)	189 (63.6)	137 (94.5)	83 (65.4)	
Tumor location, no. (%)					0.152
Right lobe of liver	278 (81.8)	241 (81.1)	125 (86.2)	108 (85.0)	
Left lobe of liver	62 (18.2)	56 (18.9)	20 (13.8)	19 (15.0)	

MVI$+$: MVI positive, MVI−: MVI negative

### 18F-FDG PET/CT examination

Biograph 64 PET/CT imager we used was made by Siemens (Germany). 18F-FDG was provided by Shanghai atomic science and technology, Ltd. The radiochemical pu-rity was more than 95%. Patients were fasted more than 6 h before examination, and their blood glucose were controlled under 11.1 mmol/l. Patients received intravenous 3.70–5.55 mbq/kg 18F-FDG according to their body mass and underwent PET/CT after lying still for 60 min. The patient firstly underwent CT scan with the following scanning parameters: tube voltage 120 kV, tube current 170 Ma; slice thickness 3.0 mm. Then, imaging with PET was performed, and a three-dimensional acquisition mode with 5–6 beds and 2.5 min/bed was used. Delayed imaging was performed in the case of diagnostic difficulties, which was performed within (120 ± 15) min after injection of 18F-FDG in 1-2 bed positions with identical scanning parameters. Attenuation correction was performed on the CT data and reconstructed by an iterative method to finally obtain transverse, sagittal, coronal CT, PET, and PET/CT fusion images. Both hospitals used the same model of PET/CT equipment for image acquisition. And we selected transverse images of PET and CT images for subsequent experiments.

### Regions of interest segmentation

**Fig. 2:**
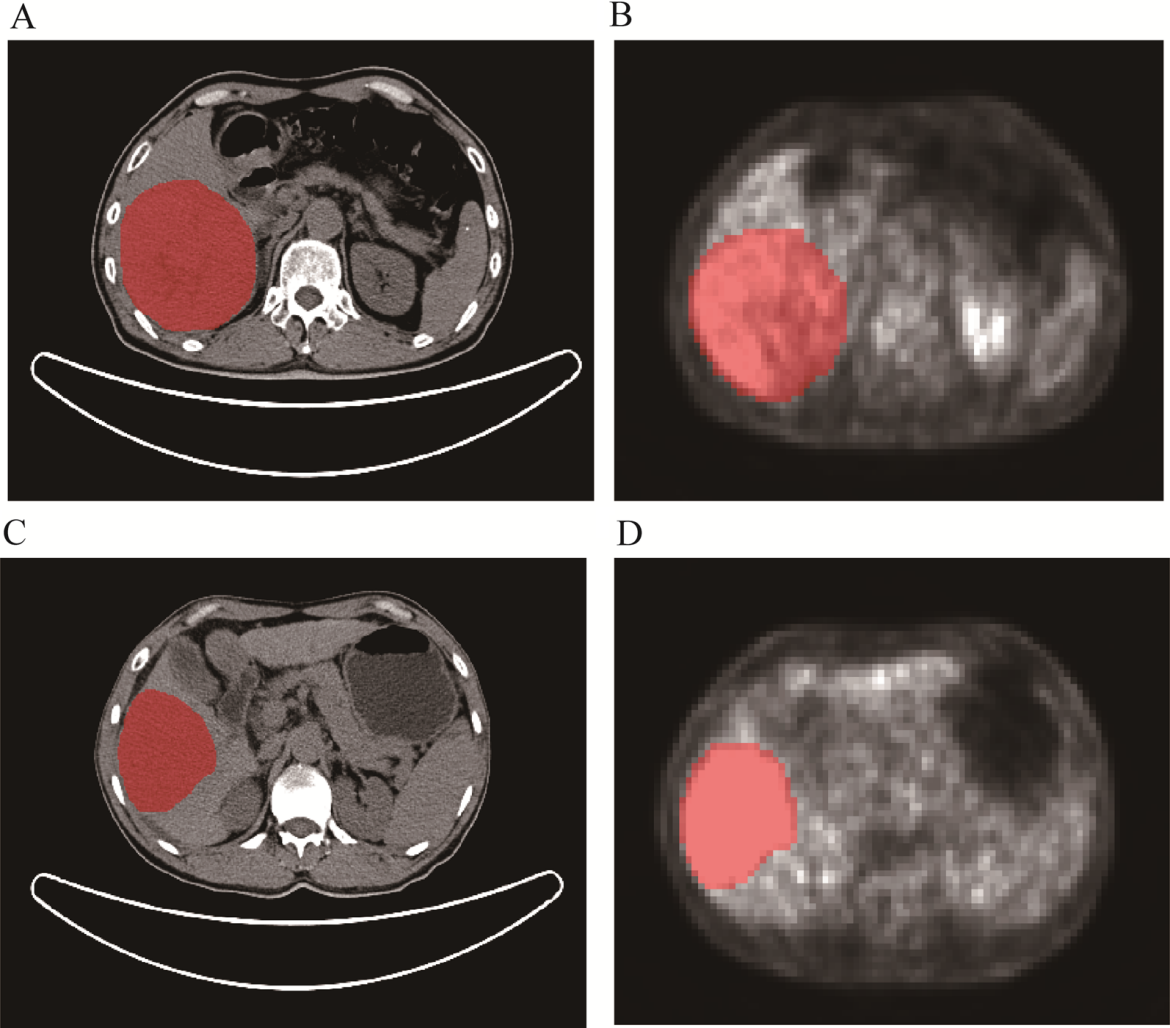
Annotation schematics of ROI were performed using ITK-SNAP software with the red portion of the figure as the manually outlined ROI. In the first row, CT (**A**) and PET (**B**) images of a 65-years-old male patient with coarse beam HCC with MVI. In the second row, CT (**C**) and PET (**D**) images for a 53-year-old male with coarse beam HCC without MVI

The tumor regions of interest (ROI) of each patient were manually drawn slice by slice by two radiologists with 5-year experience using ITK-SNAP software (available for download at http://www.itksnap.org/). As shown in Fig. 2, these were annotated schematic diagrams of ROI using ITK-SNAP software. Both radiologists were blind to the pathological results. The original image and segmentation files were stored in the format of the Neuroimaging Informatics Technology Initiative.

In order to evaluate the radiomics features extracted from the ROIs delineated by two radiologists, we calculated intraclass correlation coefficients (ICC). 150 images were randomly chosen to evaluate the inter-observer reproducibility of the radiomics feature. The ICC is a statistical measure between 0 and 1, where 0 indicates no and 1 indicates perfect reliability. Only features with ICC values equal to or higher than 0.70 were considered to be good for the subsequent research process in this study.

### Radiomics feature extraction

We extracted 465 CT radiomics features and 465 PET radiomics features from the ROI for each case, and all radiomics features were extracted by the pyradiomics package (https://pyradiomics.readthedocs.io/). The feature pool comprised 18 first-order features, 75 raw texture features, 372 wavelet-based texture features. Raw texture features included gray level co-occurrence matrix (GLCM), gray level run-length matrix (GLRLM), gray level size zone matrix (GLSZM), gray level dependence matrix (GLDM), neighbouring gray tone difference matrix (NGTDM). Numbers of GLCM-based, GLRLM-based, GLSZM-based, GLDM-based and NGTDM-based features were 24, 16, 16, 14 and 5, respectively. The features were also recomputed after different wavelet decomposition in two directions (x, y) of the original images. Performing low-pass or high-pass wavelet filter along x or y directions resulted in 4 decomposition of the original image. Consequently, we extracted 372 features by wavelet transformation of the tumor region.

### Radiomics feature selection and construction of the radiomics score

First, normalize each radiomics feature to eliminate influence caused by the numerical range differences between features. To prevent overfitting of the results, we used the least absolute shrinkage and selection operator (LASSO) regression algorithm to select the optimal contributing features group. Features with corresponding coefficients that were not-zero in the LASSO regression results were retained. Radiomics score (Rad-score) was calculated for each patient via a linear combination of selected features that were weighted by their respective coefficients.

### Development and validation of MVI-predicting nomograms

In order to analyze the significant factors affecting the prediction of MVI, univariate and multivariate logistic regression analyses were used. To identify independent clinical factors that influence the prediction of MVI, the factors with a *p* value of 0.05 or less in univariate analysis will be further analyzed using multivariate logistic regression. To prove the added value of Rad-score in MVI assessment, we established a clinical nomogram (Clin-nom) that only contains independent clinical predictors and a clinical-radiomics nomogram (C-Rad-nom) combining Rad-score with clinical predictors. We assessed and compared the performance of the established nomograms in terms of discrimination ability, calibration ability, and clinical usefulness.

### Statistical analysis

In this study, all the statistical analysis was conducted with R software (available for download https://www.r-project.org/). Categorical variables were statistically analyzed using the $\chi ^2$ test and continuous variables were analyzed using the t test. And *p* values less than 0.05 were considered statistically significant. In addition, predictive models were also established and evaluated using R software. The features selection method of LASSO was performed using the “glmnet” package. The univariate and multivariate logistic regression analyses were performed using the “glm” function. Nomograms and calibration curves were plotted used the “rms” package. The receiver operating characteristic (ROC) curves plotting and area under curve (AUC) calculation was performed using the “pROC” package. The decision curve analysis was performed with the “rmda” package.

## Results

### Clinical characteristics

Detailed clinical characteristics of cases are shown in Table 1. In 909 cases, 53.4% (485/909) were MVI positive (MVI$+$: patients were confirmed to have MVI by postoperative pathological examination) and 46.6% (424/909) were MVI negative (MVI−: patients were confirmed to have no MVI by postoperative pathological examination). MVI positivity was found in 53.4% (340/637) of tumors in the training cohort, similar to 54.3% (145/272) seen in the validation cohort (*p* = 1.000). In addition, there were no signif1icant differences between the two cohorts in other clinical characteristics. These results justified the use of the training and validation cohorts ($p>0.05$, Table 1).

### Features extraction and radiomics scores construction

**Fig. 3:**
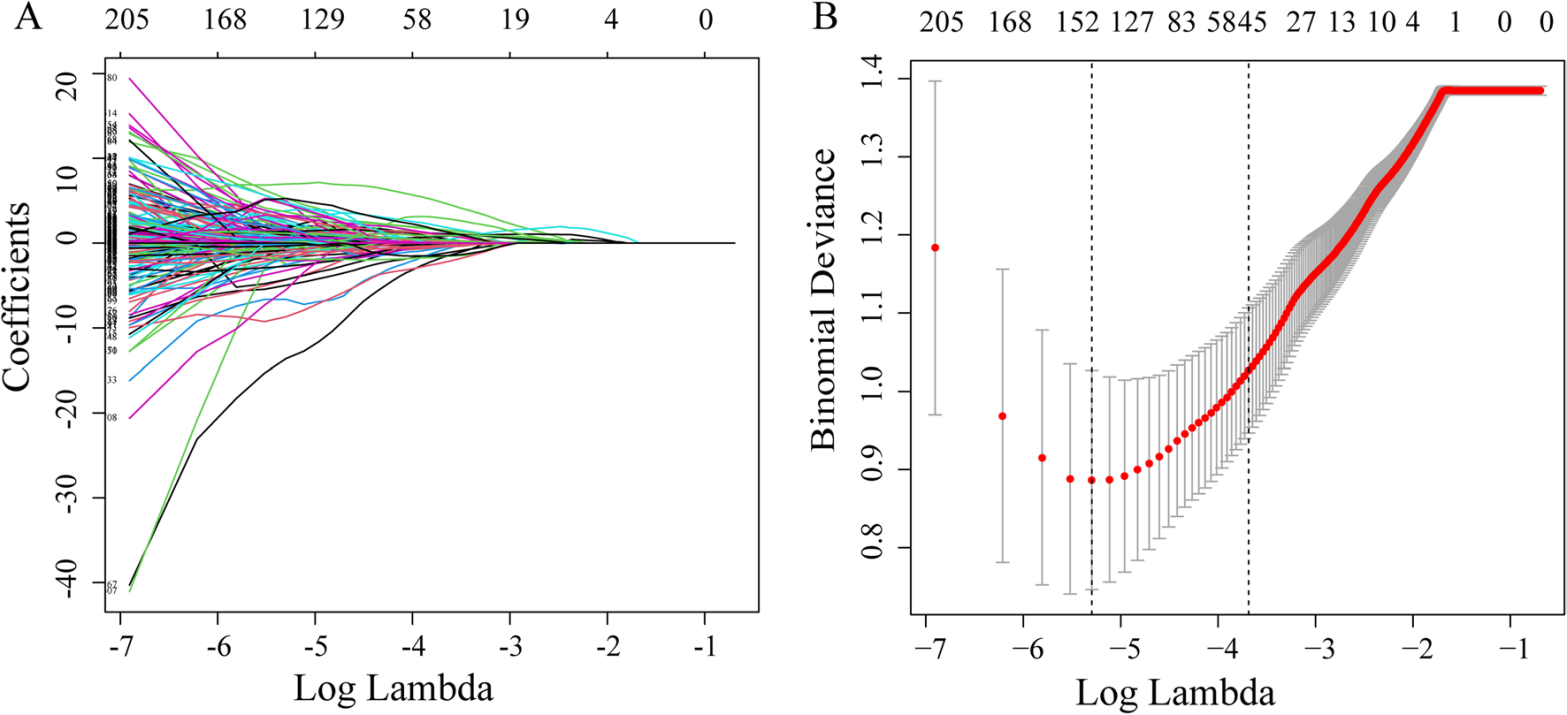
Features selection used the LASSO regression model in the features group. **A** LASSO coefficients produced by the regression analysis. As shown in **B**, $\uplambda$ = 0.0240 with log ($\uplambda$) = − 3.728 was chosen according to 10-fold cross-validation, where optimal $\uplambda$ resulted in 45 non-zero coefficients in the features group

We cascaded the features extracted from the CT image (465 radiomics features) and PET image (465 radiomics features) and our features group contained 930 radiomics features for each case. The reproducibility of radiomics feature extraction was good (ICC > 0.70). These results suggested that our radiomics feature values were highly reproducible. As shown in Fig. 3, we performed features selection using the LASSO regression model. The left figure represents the distribution of coefficients for each feature, where the coefficient profile was plotted against the log ($\uplambda$) sequence. The right showed the adjustment of parameters in the LASSO model using 10-fold cross-validation to obtain a minimum standard. The binomial deviance was plotted versus log ($\uplambda$). As shown in Fig. 3B, dotted vertical lines were drawn at the optimal values by using the minimum criteria (the left) and the 1 standard error of the minimum criteria (the right). Finally, we obtained 45 (12 features from CT and 33 features from PET) as the most significant radiomics features from the feature groups to construct Rad-score. Individual case’s Rad-score was computed through a linear combination of the selected features weighted by their respective coefficients.

### Development of MVI-predicting nomograms

Univariate and multivariate logistic regression were used to determine the relationship between the factors and MVI in HCC. As shown in Table 2, age, sex, $^{18}$F-FDG uptake status and Max/Min-TD were significantly associated with the prediction of MVI (*p* < 0.05) according to univariate logistic regression analysis. And according to multivariate logistic regression analysis, we found age, $^{18}$F-FDG uptake status and Max/Min-TD were independent predictors of MVI (*p* < 0.05).

**Table 2:** Results of the univariate and multivariate logistic regression analyses based on the training cohort

	Univariate logistic regression analysis		Multivariate logistic regression analysis	
	OR (95% CI)	*p*	OR (95% CI)	*p*
Sex	1.804 (1.083, 3.048)	0.025	0.510 (0.204, 1.492)	0.2371
Age	1.021 (1.007, 1.035)	0.004	1.083 (1.053, 1.116)	< 0.05
18F-FDG uptake status	64.190 (23.771, 263.248)	< 0.05	53.021 (13.579, 314.040)	< 0.05
Max/Min-TD	3.529 (2.111, 6.109)	< 0.05	19.655 (12.008, 34.197)	< 0.05
Tumor location	0.960 (0.643, 1.435)	0.841	–	–

**Fig. 4:**
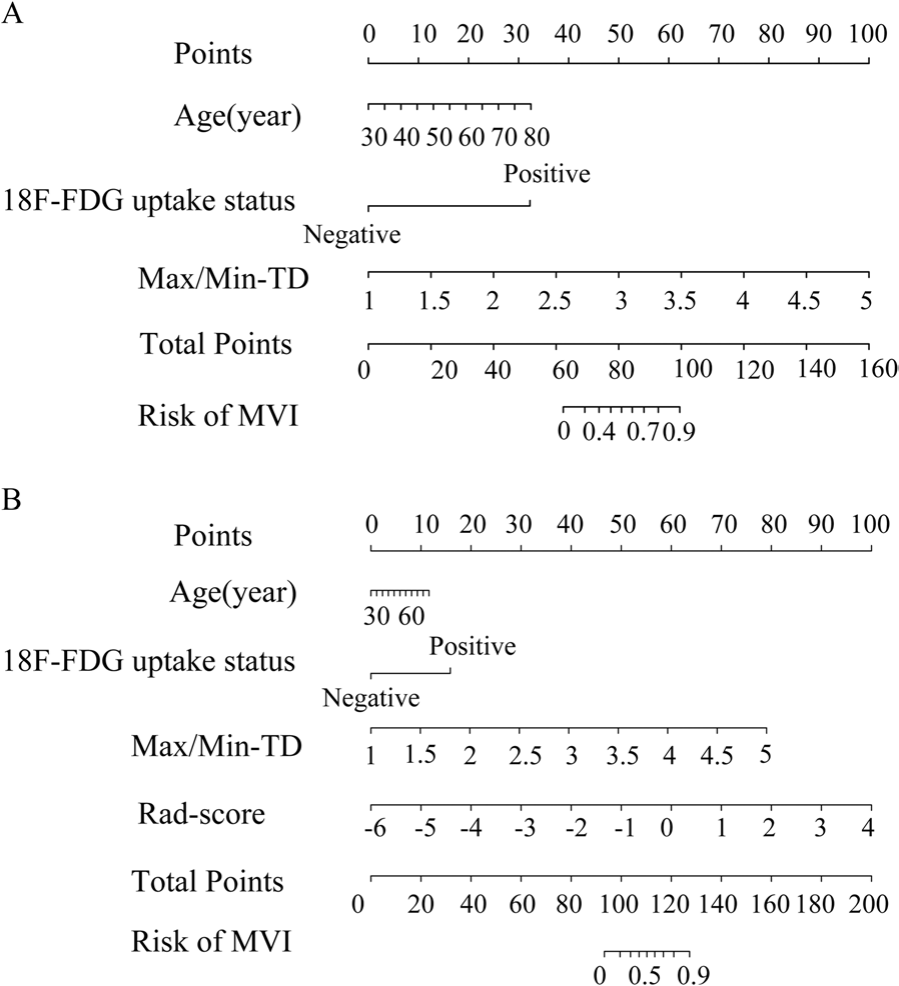
Nomograms were established to predict MVI for HCC patients. Clin-nom (**A**) and C-Rad-nom (**B**) developed from the training cohort. “Points” refers to point for the individual risk factor and add together to the “Total points”. “HCC MVI risk” was calculated according to the “Total points”

In this study, the Clin-nom was established by combining age, 18F-FDG uptake statues and Max/Min-TD. In order to verify the incremental value of Rad-score in predicting MVI status, we combined four clinical predictors with Rad-score to establish C-Rad-nom. As shown in Fig. 4A, B, Clin-nom and C-Rad-nom were established in this study.

### Validation of MVI-predicting nomograms

#### Discrimination ability

In this study, the AUC was used to identify the performance of the nomograms and Rad-score in discriminating between MVI negative and MVI positive cases (Table 3). The performance of Rad-score, Clin-nom, and C-Rad-nom in MVI prediction, AUCs were 0.903 (95% CI, 0.881–0.926), 0.941 (0.923–0.959), 0.962 (0.949–0.976) in the training cohort, and 0.806 (0.756–0.856), 0.849 (0.804–0.893), 0.890 (0.854–0.927) in the validation cohort. We performed a DeLong test to verify whether there was a significant difference among the Rad-score, Clin-nom and C-Rad-nom in MVI prediction.

**Table 3:** The AUC of Rad-score and nomograms for predicting MVI

Models	Training cohort (N = 637)			*p*	Validation cohort (N = 272)			*p*
	AUC	(95% CI)			AUC	(95% CI)		
		Lower	Upper			Lower	Upper	
Rad-score	0.903	0.881	0.926		0.806	0.756	0.856	
Clin-nom	0.941	0.923	0.959		0.849	0.804	0.893	
C-Rad-nom	0.962	0.949	0.976		0.890	0.854	0.927	
Rad-score versus Clin-nom				0.002				0.133
Rad-score versus C-Rad-nom				0.000*				0.000*
Clin-nom versus C-Rad-nom				0.000*				0.000*

*Indicated *p* < 0.0001

**Fig. 5:**
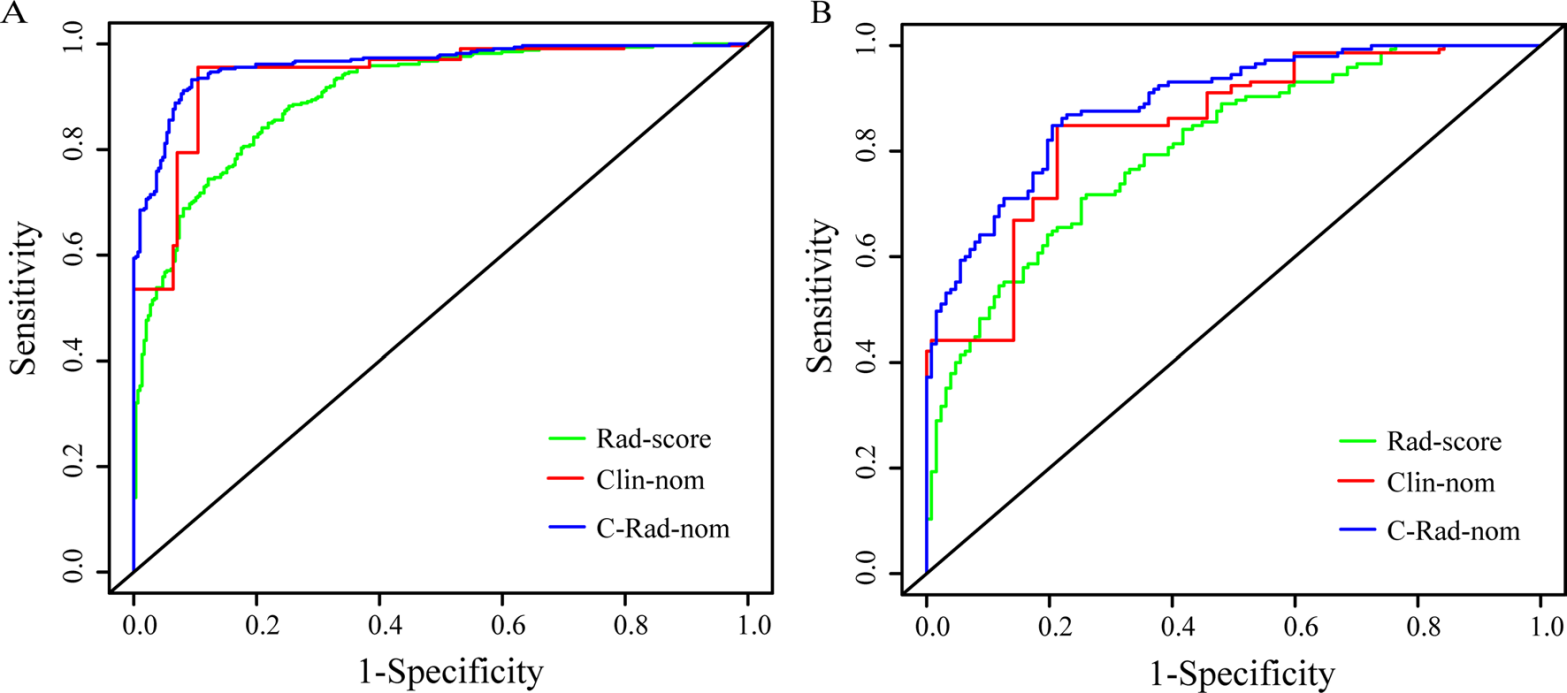
ROC curves of the Clin-nom, Rad-score and C-Rad-nom derived from the training (**A**) and validation (**B**) cohorts. The x-axis is the “1-Specifificity”, and the y-axis is “Sensitivity”. The AUCs were also presented in Table 3, respectively

According to Table 3, when Rad-score was included in Clin-nom, the performance of the Clin-nom was significantly improved in the training cohort (*p* < 0.05), and this significant improvement was also confirmed in the validation cohort, which indicates that Rad-score has incremental value in MVI prediction. The ROC curves of the Rad-score, Clin-nom, and C-Rad-nom in MVI prediction performance in the training cohort and validation cohort were depicted in Fig. 5.

#### Calibration ability and clinical usefulness

**Fig. 6:**
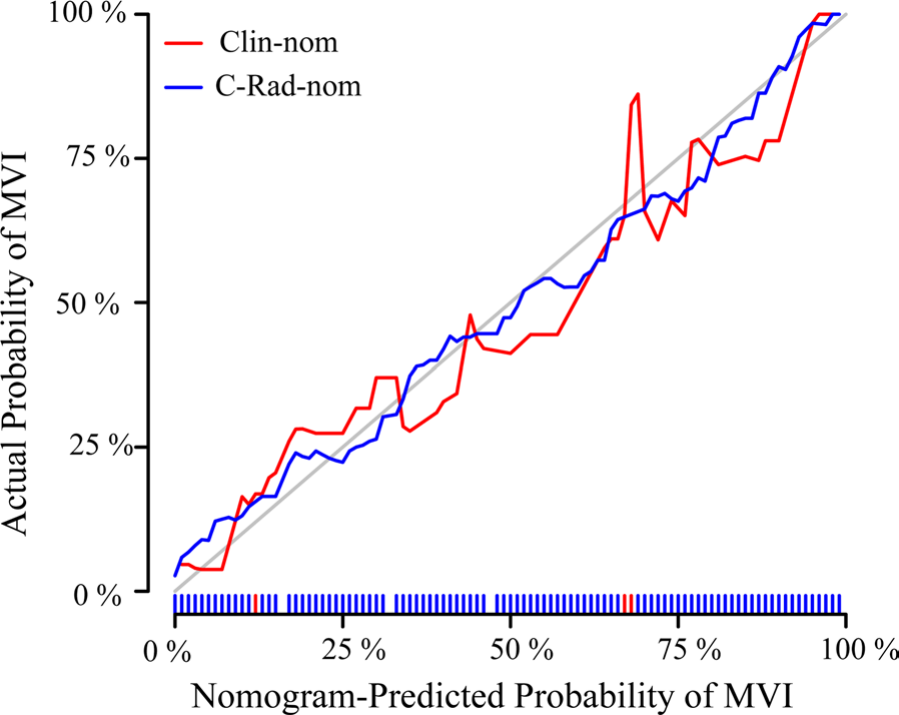
Calibration curves of the Clin-nom and C-Rad-nom generated from the validation cohort. The ordinate represents the actual probability of MVI while the abscissa represents the nomogram-predicted probability of MVI. The diagonal dashed line means that the predicted probability is equal to the actual probability and the more deviation from the diagonal indicates the greater the error of prediction

The calibration curves of Clin-nom and C-Rad-nom in the validation cohort were shown in Fig. 6. The calibration curve was used to estimate the consistentency between the nomogram-predicted probability of MVI and the actual outcomes. In Fig. 6, both nomograms showed good calibration. In contrast, the C-Rad-nom predicted probability of MVI status was consistent with the actual MVI probability, whereas Clin-nom performed worse than C-Rad-nom, with some deviation from the actual predicted probability.

**Fig. 7:**
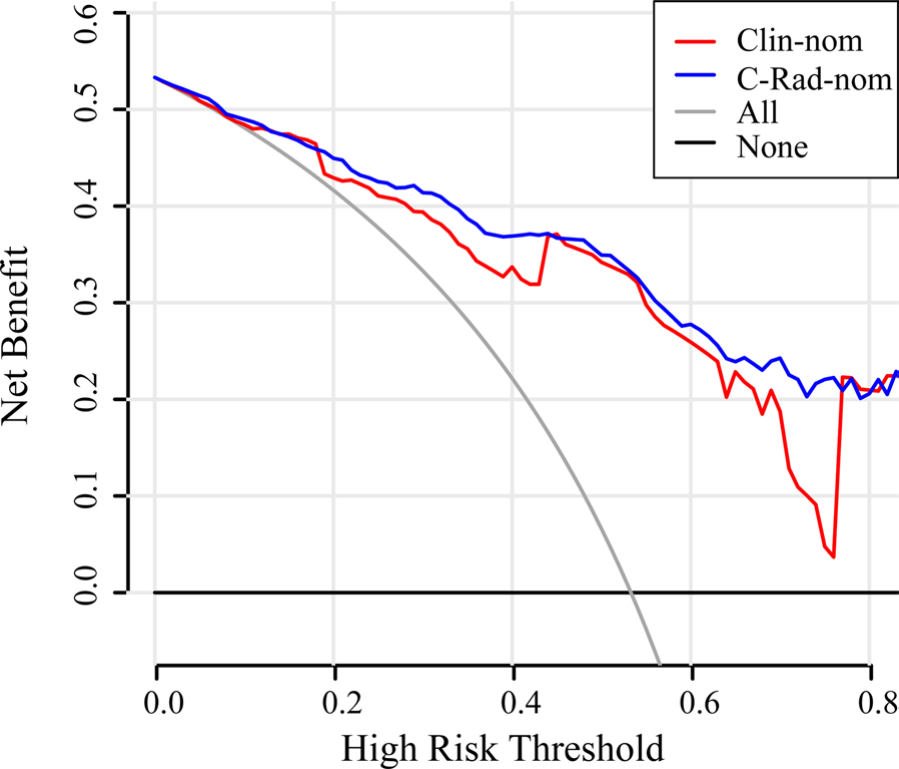
Decision curve analysis for Clin-nom and C-Rad-nom was established in the validation cohort. The abscissa shows the threshold probability, while the ordinate shows the net benefit. The gray line represents the assumption of all MVI positive cases, and the black line represents the assumption of all MVI negative cases

The decision curve analysis (DCA) evaluated the performance for the Clin-nom and C-Rad-nom in terms of clinic-pathologic application, thereby, reflecting its clinical usefulness. As the decision curve analysis shown in Fig. 7, within the probability of predicting MVI ranges of 0.1 to 0.8, there was more benefit from the Clin-nom and C-Rad-nom compared with the treat-all-patients scheme or the treat-none scheme. And if the threshold probability is greater than 0.18, the use of C-Rad-nom predicted MVI in the vast majority of cases to be more beneficial in patients using Clin-nom.

## Discussion

We have established and verified nomogram based on Rad-score for preoperative MVI prediction of HCC patients. When the Rad-score were combined with the clinical predictors in the Clin-nom, the Clin-nom performance is significantly improved (*p* < 0.05), which indicates the incremental value of the Rad-score for the prediction of individualized MVI of HCC. In addition, we combined the extracted PET and CT radiomics features to build a C-Rad-nom to facilitate the individualized prediction of preoperative MVI. The predicted calibration curve of the validation cohort was consistent with the ideal curves, and the DCA indicated that the C-Rad-nom had clinical usefulness in this study.

Tumor heterogeneity can be difficult to identify or quantify through traditional imaging tools or subjective image evaluation. Radiomics may be a useful imaging marker to improve the assessment and quantification of tumor spatial heterogeneity. The radiomics features are extracted and calculated by computer, and it is very challenging to explain the radiomics features. It is difficult for us to select biomarkers from thousands of radiomics features. The common way is to develop a multi-features parameter for outcome estimation using the radiomics technique [17, 21, 22]. In our research, we extract the radiomics features from PET/CT images, screen out the most representative radiomics features through LASSO regression, and linearly combine the corresponding coefficients to establish Rad-score, these features are indicators of tumor texture characteristics. This article confirms that Rad-score in MVI assessment has incremental value and proves that radiomics can reflect intratumoral heterogeneity.

There hasn’t reached a consensus on whether tumor size is an independent predictor in the HCC MVI assessment model. Previous studies [23–26] have reported that the tumor size of HCC was significantly different in MVI positive and MVI negative groups. The results of the previous research [27] showed that tumor diameter was only an independent factor of MVI in univariate analysis, but this result doesn’t suit multivariate analysis. In order to better describe the effect of tumor size on MVI, we proposed the clinical index of Max/Min-TD for MVI prediction. In our experiment, the diameter of the tumor was determined based on the integrated measurement of CT and PET examination images. In addition, we verified that Max/Min-TD was also significant for the prediction of MVI.

Some studies [28, 29] have shown that there is a certain correlation between the positive PET scan and MVI. The higher the malignant degree of tumor cells, the lower the degree of dephosphorylation, and more 18F-FDG deposits in the cells. Therefore, moderately and poorly differentiated HCC can usually show increased 18F-FDG uptake. In this study, 87.24% (793/909) of patients had been grade III–IV HCC, and 82.84% (753/909) of HCC showed increased 18F-FDG uptake. Additionally, research [13] has proved that 18F-FDG PET/CT positive is an independent predictor of MVI, and the specificity and sensitivity to predict MVI was 73% and 62%, respectively. In this study, 18F-FDG uptake status is an independent factor to predict MVI in HCC, which is consistent with previous research.

Nomograms incorporating multiple risk factors have been widely used to predict medical outcomes and prognosis. In this study, age, Max/Min-TD and 18F-FDG uptake status were independently related to MVI. When Rad-score was included in the Clin-nom, the Clin-nom AUC in the validation cohort was significantly increased from 0.849 to 0.890 (*p* < 0.05). In addition, the DCA shows that more patients will benefit from the C-Rad-nom rather than the Clin-nom, suggesting that the Rad-score add incremental value to the clinical usefulness of clinical predictors. Currently, models constructed with radiomics features such as ultrasound, contrast-enhanced CT, and MRI had been used to predict MVI status [17, 20, 30]. When compared with other reported models, our study demonstrated that the C-Rad-nom (0.890 [0.854, 0.927]) showed the best performance. The article by Li et al. [31] also used 18F-FDG PET/CT images for the prediction of preoperative MVI, and a predictive model for MVI status was developed using only radiomics features with an AUC of 0.692 (95% CI: 0.497–0.887) in the validation set. However, in our experiment, the MVI prediction model built using only the radiomics signature had an AUC of 0.806 (95% CI: 0.756–0.856) in the validation set. It shows that our model performs better in MVI prediction, probably because the cases they choose were at the early stage and early-stage HCC patients’ MVI status might be more difficult to predict. However, we didn’t specifically choose early-stage HCC patients in our case selection process. In conclusion, both ours and Li et al’s experimental models based on the radiomics features of 18F-FDG PET/CT images can assist physicians in preoperative HCC MVI diagnosis.

Our study has several limitations. Firstly, because of the relatively small sample size, we used each slicer of the patient as a single subject in the study. Since we are using 2D slices, we may lose some 3D features (such as volume of the tumor). Secondly, our research lacked multi-center validation. Although the C-Rad-nom proposed in this paper has been evaluated in an internal validation cohort with good results, further validation from other centers is needed to evaluate the reliability of our predicted nomograms. And the experiment was completely retrospective, so it needs to be verified by a prospective study. Finally, our clinical data were incomplete and lacked some important preoperative clinical predictors (incomplete tumor capsule, high serum $\alpha$-fetoprotein level), which may account for our decreased model performance.

## Conclusions

In conclusion, we established Rad-score based on PET/CT images of HCC patients, which can be an important factor for predicting MVI. The C-Rad-nom, which combines the Rad-score and clinical predictors, showed better performance in MVI detection compared with the Clin-nom. Thus, the C-Rad-nom had the potential to predict MVI preoperatively, enabling a more appropriate surgical plan.

## Data Availability

The datasets used and/or analysed during the current study available from the corresponding author on reasonable request.
